# The Fibrin Matrix Regulates Angiogenic Responses within the Hemostatic Microenvironment through Biochemical Control

**DOI:** 10.1371/journal.pone.0135618

**Published:** 2015-08-28

**Authors:** Ektoras Hadjipanayi, Peer-Hendrik Kuhn, Philipp Moog, Anna-Theresa Bauer, Haydar Kuekrek, Lilit Mirzoyan, Anja Hummel, Katharina Kirchhoff, Burak Salgin, Sarah Isenburg, Ulf Dornseifer, Milomir Ninkovic, Hans-Günther Machens, Arndt F. Schilling

**Affiliations:** 1 Department of Experimental Plastic Surgery, Clinic for Plastic and Hand Surgery, Klinikum rechts der Isar, Technische Universität München, D-81675, Munich, Germany; 2 Department of Plastic, Reconstructive, Hand and Burn Surgery, Bogenhausen Hospital, 81925, Munich, Germany; 3 German Center for Neurodegenerative Diseases (DZNE), Munich, Germany; 4 Neuroproteomics, Klinikum rechts der Isar, Technische Universität München, Munich, Germany; 5 Department of General Paediatrics, Neonatology and Paediatric Cardiology, University Children‘s Hospital Düsseldorf, 40225, Düsseldorf, Germany; 6 Cambridge University Department of Paediatrics, Cambridge University Hospitals NHS Foundation Trust, Cambridge, United Kingdom; 7 Center for Applied New Technologies in Engineering for Regenerative Medicine (Canter), Munich, Germany; Center for Cancer Research, National Cancer Institute, UNITED STATES

## Abstract

Conceptually, premature initiation of post-wound angiogenesis could interfere with hemostasis, as it relies on fibrinolysis. The mechanisms facilitating orchestration of these events remain poorly understood, however, likely due to limitations in discerning the individual contribution of cells and extracellular matrix. Here, we designed an *in vitro* Hemostatic-Components-Model (HCM) to investigate the role of the fibrin matrix as protein factor-carrier, independent of its cell-scaffold function. After characterizing the proteomic profile of HCM-harvested matrix releasates, we demonstrate that the key pro-/anti-angiogenic factors, VEGF and PF4, are differentially bound by the matrix. Changing matrix fibrin mass consequently alters the balance of releasate factor concentrations, with differential effects on basic endothelial cell (EC) behaviors. While increasing mass, and releasate VEGF levels, promoted EC chemotactic migration, it progressively inhibited tube formation, a response that was dependent on PF4. These results indicate that the clot’s matrix component initially serves as biochemical anti-angiogenic barrier, suggesting that post-hemostatic angiogenesis follows fibrinolysis-mediated angiogenic *dis*inhibition. Beyond their significance towards understanding the spatiotemporal regulation of wound healing, our findings could inform the study of other pathophysiological processes in which coagulation and angiogenesis are prominent features, such as cardiovascular and malignant disease.

## Introduction

Hemostasis and angiogenesis are two closely interlinked physiological processes that upon vascular injury harmoniously operate to re-establish the microcirculation to its former state [1]. Effective coagulation is necessary directly after injury to prevent excessive bleeding. Immediate (i.e. premature) initiation of angiogenesis would be counterproductive at this stage, since newly formed vessels are fragile and unstable [2]. It is therefore not surprising that the two processes are tightly controlled, to ensure that angiogenesis is only triggered once hemostasis has been safely completed. This is evident during wound healing, where angiogenesis does not begin before three days after wounding [1,3]. Furthermore, it is reflected in the fact that various hemostatic factors (e.g., Prothrombin-derived fragments 1 and 2, Fibrinogen E fragment, Plasminogen fragment/Angiostatin) possess anti-angiogenic activity [4,5]. While it is generally accepted that these factors, and their induced cellular responses, are important components of a greater regulatory mechanism [4,5], an overarching theory which could explain how hemostasis and angiogenesis are coordinated, in a spatiotemporal manner, is still lacking.

It is intriguing that post-wound hemostasis and angiogenesis occur within the same biomaterial; fibrin. Following activation of coagulation, the fibrin matrix entraps platelets at the site of injury, forming a hemostatic plug, that is gradually replaced by capillary-rich granulation tissue, and eventually collagen, leading to restoration of the original extracellular matrix (ECM) architecture [1]. The classic wound healing model acknowledges an active role for cells which influence downstream cell behavior through protein factor signaling, and a primarily passive role for the ECM that provides a scaffold for migrating/proliferating cells [1,6,7]. The concept that the fibrin matrix may additionally serve as a sustained release reservoir for endothelial cell (EC) growth factors has previously been proposed [1,5,8], but the mechanism(s) facilitating this function, as well as its effects on the temporal orchestration of the aforementioned events remain ambiguous.

**Fig 1:**
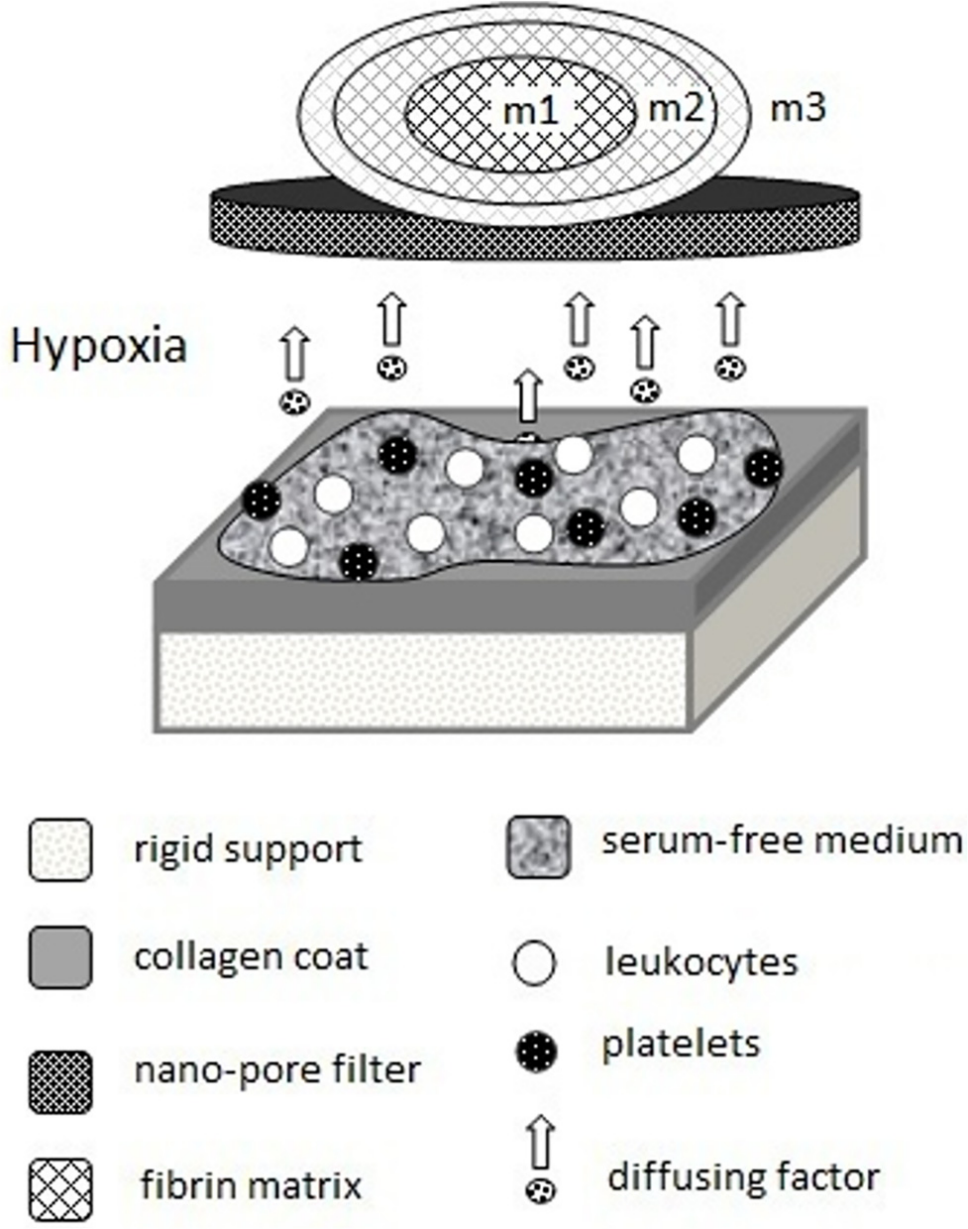
Hemostatic Components Model (HCM). Peripheral blood cells (platelets and leukocytes) are seeded at their naturally-occurring ratio onto a collagen-coated substrate, which mimics exposed collagen in the injured vascular wall. Cells are cultured in serum-free medium under hypoxia (3%O_2_) at 37°C, while released/produced protein factors diffuse through a nano-porous filter and are sampled simultaneously within an exogenous fibrin matrix of varying mass (m). Cell-matrix contact is prevented through the filter. The model simulates the hemostatic microenvironment, while enabling assessment of the function of the fibrin matrix as protein factor-carrier, independently of its role as cell-scaffold.

Perhaps, the main difficulty encountered while trying to discern the interplay between cellular and matrix components is their inherent association, as the early clot comprises platelets within a fibrin network that is subsequently populated by other cell types as the clot matures [1]. It is therefore not possible to differentially assess the role of both entities through observations of the coagulation/wound healing process *in vivo*, or by using a simply reconstructed *in vitro* wound model. Instead, this could be achieved by using an *in vitro* model of hemostasis that simulates the functional association of the cellular/matrix components, while maintaining a physical separation between them. Here, we designed such a Hemostatic Components Model (HCM) (Fig 1), and used it to investigate the role of the fibrin matrix exclusively as protein factor-carrier, independently from its well-described role as cell-scaffold. It is already known that fibrin(ogen) binds many of the pro- and anti-angiogenic factors (e.g. VEGF [8,9], FGF [8,10], PDGF [8], PF4 [11], TSP1 [5]) that are released following coagulation. Indeed, the ability to bind and release these factors offers the possibility for both contact-mediated, as well as long-range regulation of cellular responses, a prerequisite for generating spatiotemporally-defined angiogenesis [7,12,13]. It is then possible that, through its factor-carrier function, the matrix can modulate its angiogenic conductivity as cell-scaffold.

Given that effective hemostasis relies on the formation of a stable clot, while vascularization of the fibrin matrix is dependent on controlled fibrinolysis (which enables EC invasion) [5,14,15], we hypothesized that the hemostatic/angiogenic switch operates through changes in the mass of the matrix (which determines the total fibrin concentration within the wound under a constant wound volume, i.e. before wound contraction sets in), and consequently in the relative amounts of pro- and anti-angiogenic factors that are bound/released by the matrix into the hemostatic microenvironment. Our findings lead us to propose a mechanism of matrix-dependent biochemical control, through which the fibrin matrix can seamlessly perform its dual role, initially as an anti-angiogenic hemostatic barrier and later as an angiogenic scaffold.

## Materials and Methods

### Hemostatic Components Model (HCM)

All blood donors provided written informed consent as directed by the ethics committee of the Heinrich Heine University, Düsseldorf, Germany, which approved this study. The buffy coat was isolated from 10ml peripheral blood, by centrifugation in EDTA-Vacutainer tubes (BD, Germany) at 3000rpm/4°C for 15min, and reconstituted in 10ml serum-free (SF) medium (AIM V, Invitrogen, Germany). 1ml blood cell (BC)/SF-medium mixture was added to type I collagen-coated wells (area ~10cm^2^) containing 2ml SF medium. Fibrin matrices of varying protein mass were formed by combining 0.5, 1 or 1.5ml fibrinogen solution (fibrinogen 80mg/ml, aprotinin 3000KIU/ml, factor XIII 10–50IU/ml, fibronectin 2–9mg/ml) (Baxter, Germany) with an equal volume of thrombin (500 IU/ml)/Ca^+2^ solution (Baxter, Germany), in cell-culture inserts with a 1μm pore PET membrane (BD, Germany). Where necessary, the total volume per insert was supplemented accordingly with SF medium to 3ml. Inserts were then transferred into the collagen-coated wells. Culture was carried out under hypoxia (3% O_2_) within a 37°C/5% CO_2_ incubator (Fig 1).

### Measurement of oxygen tension in blood culture

Peripheral venous blood (WBC: ~5x10^9^/L) was collected from four 25yr old healthy (BMI = 23.6±2.5 Kg/m^2^), non-smoker subjects. For monitoring O_2_ tension, blood was added to 24-well plates (area ~2cm^2^) having a sensor spot at the bottom (OxoDish, PreSens, Germany), at the following blood incubation volume (BIV): 0.5, 1, 1.5 and 3ml. SF-medium (1ml) was tested as control. The sensor spot contains a luminescent dye which is excited by the SensorDish Reader (resolution = ±0.4% O_2_ at 20.9% O_2_, precision = ±1% O_2_ at 20.9% O_2_, drift< 0.2% O_2_ within one week) placed below the multidish, while the dye's luminescence lifetime, which depends on O_2_ partial pressure of the medium, is detected through the transparent bottom. Wells were airtight sealed with Parafilm. Cell culture was carried out within a normoxic incubator (37°C/5% CO_2_) for 7 days. O_2_ tension was measured for 30 min every day for 7 days. Four samples were tested per subject for each BIV.

### Analysis of protein factors in culture supernatants and matrix releasates

#### Proteomic analysis with mass spectrometry

Following 7 days HCM culture, fibrin matrices (2cm^3^) were removed from inserts and added to 1ml fresh SF medium (albumin-free medium was used to reduce interference with peptide detection, see S1 Table), then centrifuged at 3000rpm/4°C for 15min, to obtain releasates. Absolute total protein concentration of media samples (~4 μg/μl) was determined with BCA assay. 150μg of each sample were separated on a 12% SDS page gel which subsequently was cut into 16 bands. Tryptic in gel digestion was performed for each gel band. Peptide analysis was performed on an Easy nLC nanoflow HPLC 1000 system (Proxeon) connected to a LTQ-Velos Orbitrap (Thermo Fisher Scientific). Peptides were separated online by reverse phase chromatography using in-house made 30cm columns (New Objective, FS360-75-8-N-S-C30) packed with C18-AQ 2,4 μm resin (Dr. Maisch GmbH, Part No. r124.aq). An 80min gradient (5% to 40%) at a flow rate of 250nl/min was used. The measurement method consisted of an initial FTMS scan recorded in profile mode with 30.000m/z resolution, a mass range from 300–2.000m/z and a target value of 1.000.000. Subsequently, collision-induced dissociation (CID) fragmentation was performed for the 15 most intense ions with an isolation width of 2Da in the ion trap. A target value of 10.000, enabled charge state screening, a monoisotopic precursor selection, 35% normalized collision energy, an activation time of 10ms, wide band activation and a dynamic exclusion list with 30s exclusion time were applied. Data of two biological replicates of the fibrin releasates were analyzed with the MaxQuant suite (version 1.5.0.12). Protein identification was performed using the integrated Andromeda search algorithm. First search, mass recalibration and main search of tryptic peptides were performed using a human Uniprot database downloaded 08/21/2012 (86749 entries) allowing for N-terminal acetylation and oxidation of methionine as variable modifications and carbamidomethylation of cysteine as fixed modification. Two missed cleavages were allowed. Peptide as well as protein false discovery rate was set to 0.1%. Mass accuracy was set to 20 ppm for the first search and 5 ppm for the main search. Intensity based absolute quantitation (IBAQ) of proteins was performed with the IBAQ algorithm implemented in MaxQuant.

#### Angiogenesis proteome assay

Fibrin matrix releasates, obtained as described above, were analyzed with Angiogenesis Proteome Profiler array (R&D, USA), according to manufacturer’s instructions. SF medium was tested as negative control. Quantification of relative factor levels (sample signal/reference signal) was carried out by image analysis of scanned x-ray film images (3,4 and 5 min exposures) using an imaging software (Image J, NIH, USA). An averaged background signal was subtracted from the average pixel density of each pair of duplicate spots. Three samples were tested.

#### Elisa

For testing the effect of clot size on Vascular Endothelial Growth Factor (VEGF) expression, clot supernatants were obtained from 7 day coagulated blood cultures (37°C/5% CO_2_) with a BIV = 1, 1.5 and 3ml, and from 1–5 day coagulated blood cultures with a BIV = 3ml, and analyzed for VEGF by ELISA (R&D, USA), according to manufacturer’s instructions. Three samples were tested per experimental condition.

For testing the effect of hypoxia on VEGF expression, peripheral blood (5ml) was collected into EDTA-Vacutainer tubes (BD, Germany) from 48 healthy subjects; 20 males (age = 39±3.6yrs, BMI = 26.9±0.9Kg/m^2^) and 28 females (age = 34.7±2.2yrs, BMI = 23.2±0.7Kg/m^2^). Blood from each subject was mixed with 5ml SF-medium in the EDTA-Vacutainer tubes and aliquoted into four 6-well plates (2.5ml/well). Plates were placed in a 37°C/5% CO_2_ normoxic or hypoxic (3% O_2_) incubator, and cultured for 7 days (blood remained anticoagulated over this period), after which supernatants were sampled from wells and tested with ELISA for VEGF (R&D, USA). Two samples from each subject were tested per experimental condition (normoxia/hypoxia).

For measuring factor levels in fibrin clot releasates, plasma (1ml) was obtained after 2, 4, 7 or 8 days culture of anticoagulated blood at BIV = 3ml, as indicated, and mixed with various ratios of fibrinogen (80mg/ml):thrombin (500 IU/ml)/Ca^+2^ solution; 0:0.2ml, 0.1:0.1ml, 0.2:0.2ml, resulting in clots of total (i.e. wet) mass = 100, 200 and 300mg, respectively (note; when fibrinogen was added, the amount (8 or 16mg) exceeded that present in 1ml plasma, i.e. these clots comprised mostly exogenous fibrinogen). In other experiments, plasma (2ml) was obtained after 1hr incubation of anticoagulated blood (5ml) in type I collagen-coated wells, and mixed with 1.5ml thrombin/Ca^+2^ solution to form fibrin clots (v = 1cm^3^) or added to type I collagen matrices (v = 1cm^3^, d = 4 mg/cm^3^). Fibrin clots and collagen matrices were incubated in plasma for 2, 4, 8 or 24h, as indicated, then removed and centrifuged in 2ml fresh SF medium at 2000rpm/4°C for 15min to obtain releasates. For measuring the rate of factor release, releasates were obtained by incubating fibrin clots in fresh SF medium over 2, 4, 8, 12 and 24h on a rocking platform.

For measuring factor levels in cell-free fibrin matrix releasates, matrices were obtained after 7 days HCM culture and added to 2ml fresh SF medium, then incubated for 12h on a rocking platform, before sampling the releasate. In other experiments, cellulose-based hydrogel (Hydrosorb, Hartman, Germany) or polyhexanide hydrogel matrices were cultured in the HCM, and processed in the same manner as fibrin matrices. Clot/matrix releasates were analyzed for VEGF, Thrombospondin 1 (TSP1) and Platelet Factor 4 (PF4) by ELISA (R&D, USA). Three samples were tested per experimental condition.

### Test of the angiogenic potential of cultured clot and fibrin matrix releasates

Clots were obtained from 10ml coagulated blood samples and cultured in the presence of blood serum (3ml) on type I collagen matrices (v = 5cm^3^, d = 4mg/cm^3^) at 37°C for 4 days. After culture, clot releasates were obtained by centrifugation at 2000rpm/4°C for 15min and collection of the serum supernatant. Fibrin matrix releasates were obtained following 7 days HCM culture, as described above. Releasates were tested in the following assays; *Directional EC migration assay*: chemotactic migration of human umbilical vein ECs (HUVECs; CellSystems, Germany) through a matrigel-coated PET membrane over 24h at 37°C/5%CO_2_ was tested using the BD BioCoat Angiogenesis system (BD, USA), which allows exclusive visualization of invasive cells labeled with DilC_12_ Fluorescent Dye (FluoroBlok, BD, USA). An inverted fluorescence microscope was used to view each well and quantification of fluorescence intensity was carried out with an imaging software (Image J, NIH, USA). SF medium served as control. *Tube formation assay*: HUVECs were seeded on factor-reduced matrigel (BD, Germany), at 10x10^3^ cells/well and 50μl of releasate or control media (SF media without or with VEGF (90pg/ml) were tested as negative and positive controls, respectively) were added per well (μ-Slide Angiogenesis, Ibidi, Germany). For blocking experiments, 40μl rabbit anti-human anti-PF4 antibody (3μg/ml) (Abcam, Germany) was added per 1ml of releasate. After 16h culture at 37°C/5%CO_2_, cells were stained with Calcein AM (PromoKine, Germany), and tube formation was observed with phase contrast and fluorescence microscopy. Assessment of the extent of capillary-like network formation was carried out by counting the number of tubules and nodes (a node was defined as the point of intersection of two or more tubules). *Sprouting assay (aortic ring model)*: Aortic rings were dissected from female adult mice, underwent overnight serum starvation in opti-MEM Reduced Serum medium (Life Technologies, Germany) and embedded into matrigel bilayer matrix (50μl/layer in 96-well plates). Releasates and control SF media without or with VEGF (90ng/ml) were added to the rings (150μl/well), before culturing them in 37°C/5%CO_2_. Medium change was carried out every 3 days, while rings were observed with phase contrast microscopy at 1, 3, 5 and 7 days and photographed, with all 4 quarters per ring analyzed for sprouting (formation of structures of connected ECs that were attached at their base to the ring). For all assays, at least four samples were tested per experimental condition, with a minimum of 4 fields analyzed per sample.

### Statistical Analysis

For each experimental condition n ≥ 3 was used. Data is expressed as mean ± standard deviation or mean ± standard error, as noted. Statistical analysis was carried out using Student’s independent t-test where a maximum of 2 groups was compared or oneway ANOVA accompanied with multiple comparison tests for analysis of more than 2 groups, using SPSS 14 software. The probability of a type one error was set to 5% (α = 0.05), unless noted otherwise.

## Results

### Dependence of ambient O_2_ tension and angiogenic factor expression on clot size

**Fig 2:**
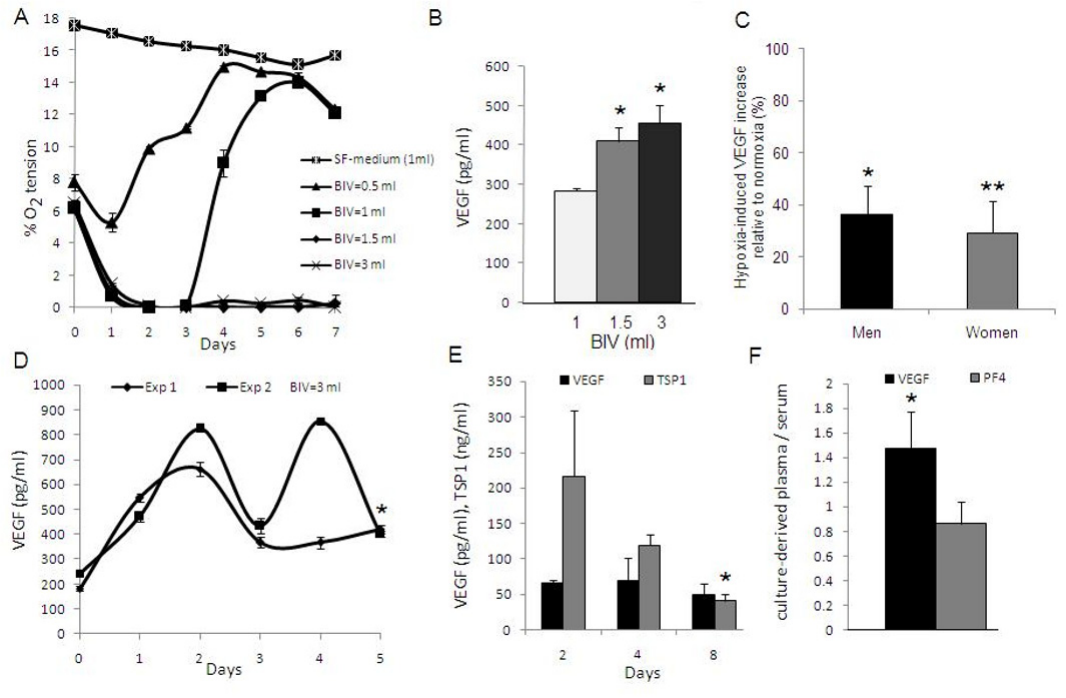
Hypoxia potentiates coagulation-mediated angiogenic signaling. A) Plot of the temporal profile of pericellular O_2_ tension in coagulated blood cultures, carried out in sealed chambers (bottom area~2cm^2^) at 37°C for 7 days, for four BIV (blood incubation volume) values (day 0 O_2_ tension corresponds to that in peripheral venous blood). SF-medium was tested as control. Data shown are typical for a young, healthy subject (n = 4). B) Plot of clot supernatant VEGF concentration vs. BIV after 7 days culture in the same setup, *p<0.01 compared to BIV = 1ml (n = 3). C) Plot comparing the normoxia- vs. hypoxia-induced VEGF expression in anticoagulated blood, obtained from male (n = 20) and female (n = 28) subjects, and cultured at 37°C under normoxia or hypoxia (3% O_2_) for 7 days. Data are presented as % increase in VEGF concentration in hypoxic culture supernatant, relative to the normoxic baseline. Error bars represent s.e.m., *p<0.01, **p<0.001 corresponds to hypoxic vs. normoxic culture mean VEGF concentration (n = 2 per subject, per condition). D) Plot of the temporal profile of clot supernatant cumulative VEGF concentration in coagulated blood cultures with a BIV = 3ml, carried out in 37°C sealed chambers over 5 days, *p<0.001 compared to day 0 levels. Data shown are from two independent experiments, carried out on blood from same subject (n = 3 per exp.). E) Plot of VEGF and TSP1 concentration in releasates of cell-free fibrin clots that were formed and incubated for 24h in plasma derived from anticoagulated blood, after it had been cultured at 37°C, BIV = 3ml for 2, 4, or 8 days, *p<0.05 compared to day 2 levels (n = 3). F) Plot of the ratio of VEGF and PF4 concentration in plasma, derived from 5 day anticoagulated blood cultures carried out on collagen-coated substrates under hypoxia (3% O_2_) at 37°C, relative to serum concentration, *p<0.05 corresponds to plasma vs. serum concentration (n = 3). Unless otherwise noted, error bars represent s.d.

Size and composition of the clot change during progression of wound healing through alterations in fibrin content and number of resident blood cells (BCs) [16]. Under conditions of limited O_2_ supply, ambient O_2_ tension within and around the clot, and consequently O_2_-regulated angiogenic factor expression [17], will inevitably be defined by BC density since this will determine the net cellular respiration. Hence, the fibrin matrix could indirectly influence coagulation-mediated angiogenic signaling through its function as cell-scaffold. To test this hypothesis we first studied the effect of increasing clot volume and consequently BC areal density on the temporal profile of pericellular O_2_ tension in coagulated blood cultures, by varying the blood incubation volume (BIV) within sealed chambers (i.e. under conditions of limited O_2_ availability). Fig 2A compares the O_2_ tension profile for four BIV values; 0.5, 1, 1.5 and 3ml, corresponding to a white blood cell (WBC) areal density of ~1.25x10^6^, ~2.5x10^6^, ~3.75x10^6^ and ~7.5x10^6^ cells/cm^2^, respectively. O_2_ tension in cultures with a BIV of 0.5ml never fell below 5%, as the cells likely quickly adapted to the reduced O_2_ supply by decreasing O_2_ consumption [18,19]. The same effect could be observed in BIV of 1ml, while in cultures with a BIV of 1.5 and 3ml O_2_ tension was maintained below 1% for up to 7 days.

We next examined whether an increase in clot size, and the resulting prolongation of pericellular hypoxia, also correlated with increased BC pro-angiogenic factor expression, by measuring VEGF levels in clot supernatants obtained from 7 day coagulated blood cultures with a BIV of 1, 1.5 and 3ml (note; supernatant factor concentration is initially independent of BIV as cell number increases proportionally with volume). While there was no significant difference between 1.5 and 3ml BIV (prolonged hypoxic plateau), VEGF concentration in these cultures was approx. 1.5 fold higher than in cultures with a BIV of 1ml (short hypoxic plateau) (p<0.01) (Fig 2B). To study the population-dependent statistical variation of the hypoxia-induced VEGF upregulation, independent of an accompanying platelet-derived VEGF release, anticoagulated blood was obtained from 48 age- and BMI-matched healthy subjects, and cultured under controlled hypoxia (3% O_2_) or normoxia for 7 days. As shown in Fig 2C, hypoxia induced a 30–50% increase in supernatant VEGF concentration relative to the normoxic baseline.

Since cellular angiogenic factor expression exhibits adaptation under prolonged hypoxic stress [20,21], we sought to characterize the temporal profile of VEGF production within hypoxic clots. Coagulated blood cultures were carried out within sealed chambers at a BIV of 3ml (pericellular O_2_<1% after 24h), while VEGF levels in clot supernatants were measured over 5 days. VEGF cumulative concentration rose in the first 2 days, above the platelet-derived baseline (~200 pg/ml), reaching a peak of ~600–800 pg/ml (Fig 2D). Thereafter, levels dropped, indicating a net decrease in the balance of VEGF production and degradation under prolonged hypoxia. While some variation in the expression profile was encountered between experiments (blood obtained from same subject), day 5 levels were always found to be ~50% higher than baseline (p<0.001), as expected from the results above.

It has been reported that hypoxia has an opposing effect on the expression of pro- and anti-angiogenic factors, such as VEGF and TSP1[22,23]. We also previously showed that expression of TSP1, an angiogenic inhibitor secreted by activated platelets and monocytes, is downregulated over time in hypoxic blood culture [21]. Here we tested whether this temporal shift in the biochemical balance was replicated within fibrin clots. Blood was cultured in an anticoagulated state (platelet-derived TSP1 release was prevented to reduce background noise) at a BIV of 3ml for 2, 4 and 8 days (pericellular O_2_<1% after 24h), after which plasma was obtained and combined with fibrinogen-thrombin/Ca^2+^ to induce clot formation. Cell-free clots (wet mass = 300mg) were incubated in plasma for 24h, before being centrifuged in fresh medium to produce releasates. As can be seen in Fig 2E, there was a progressive reduction in TSP1 clot releasate level, in contrast to VEGF whose level remained stable. Therefore, beyond providing simple factor storage, fibrin clots appeared to have the capacity to register relative changes in hypoxia-regulated pro- and anti-angiogenic factor expression.

### Hemostatic Components Model (HCM) validation

HCM culture (Fig 1) enables protein factors that are released/produced by isolated platelets and leukocytes (reconstituted in serum-free medium at their natural ratio), through collagen-mediated activation and hypoxia-induced stimulation, to be simultaneously sampled within an exogenous cell-free fibrin matrix. We validated this model, with respect to our previous data, by comparing VEGF levels in plasma obtained from 5 day old (anticoagulated) blood cultures, carried out under hypoxia (3% O_2_) on collagen-coated substrates, to serum VEGF levels (where levels are mainly defined by platelet factor release upon coagulation). Serum levels of PF4 were in the range reported in literature (7030±310 ng/ml [24]). PF4 levels in culture-derived plasma (6070±1280 ng/ml) were not significantly different from serum (Fig 2F), thereby indirectly confirming collagen-mediated platelet activation in our culture system. These levels of PF4 were also significantly higher (p<0.01) than those measured in plasma obtained after 1h incubation of (anticoagulated) blood on collagen-coated substrates (1205±540 ng/ml), indicating a continuous factor release from platelets over 5 days when collagen was used as activator, in agreement with the literature [25]. In contrast, VEGF concentration was ~50% higher in 5 day culture-derived plasma (478±73 pg/ml) than in serum (327±42 pg/ml) (p<0.05), reflecting the additional hypoxia-induced VEGF production, in agreement with our measurements of VEGF levels in 5 day clot supernatants.

### The fibrin matrix functions as carrier of factors released in the hemostatic microenvironment

**Fig 3:**
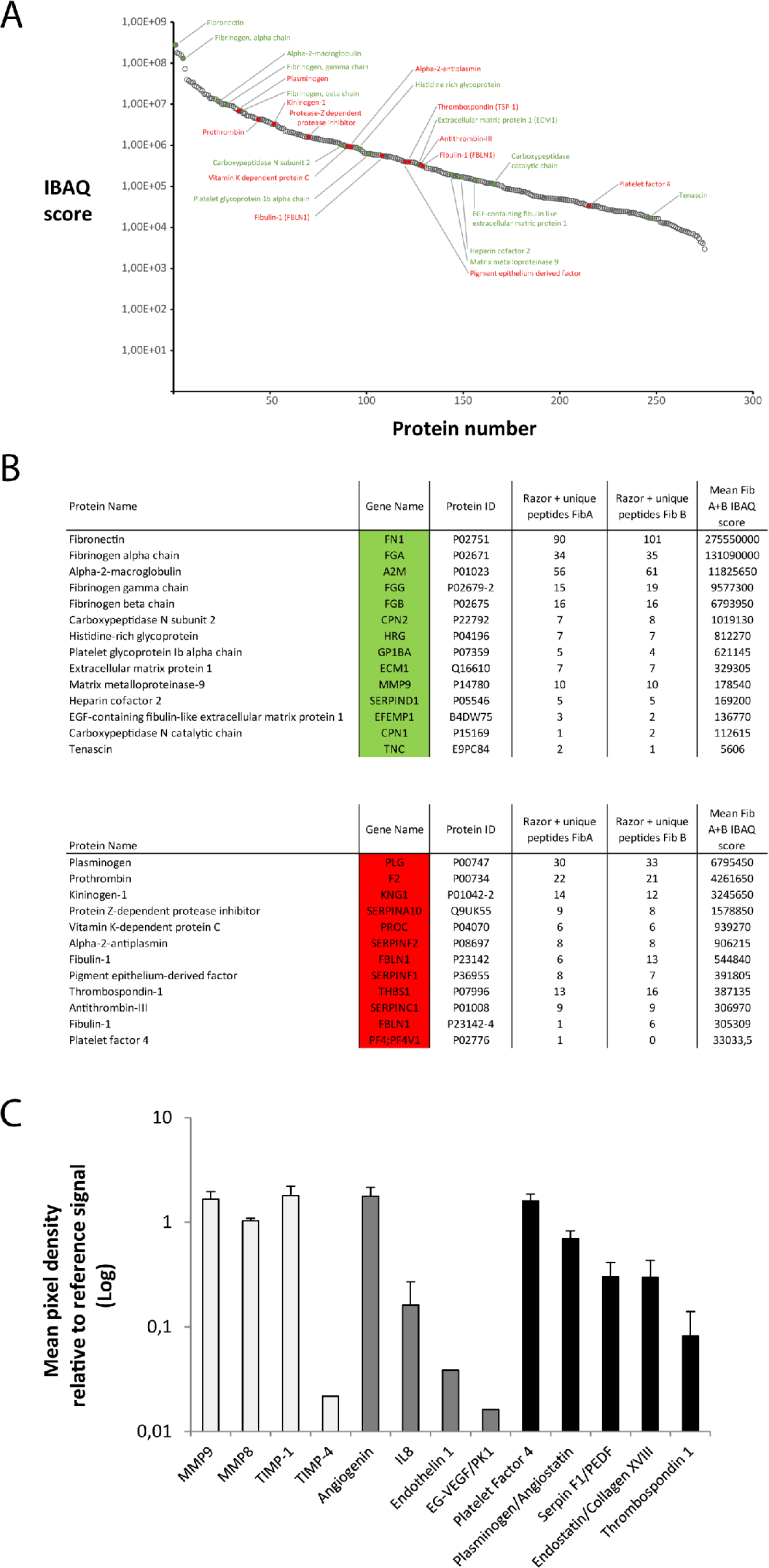
The fibrin matrix functions as carrier of pro- and anti-angiogenic factor proteins. A) Plot of all proteins identified in fibrin matrix releasates, obtained after 7 days HCM culture, in at least one of two biological replicates with at least one unique peptide via mass spectrometry. Proteins are sorted from high (left) to low (right) abundance according to their IBAQ score. Pro-angiogenic proteins are highlighted in green and anti-angiogenic proteins are highlighted in red. B) Tables of pro-angiogenic proteins marked in green and anti-angiogenic proteins marked in red. Under gene names the gene symbol can be found. Under protein identifiers the uniprot identifier of the respective protein is listed. Unique Peptides FibA and FibB indicate the identified peptides of each protein for the two biological replicates (note; this does not indicate relative protein abundance). Table with complete proteomic analysis and unprocessed output files can be found under supplementary data (S2 and S3 Tables). C) Plot showing the profile of angiogenesis-related proteins, as analysed by proteome profiler array, in the same releasates. White, grey and black bars show matrix-remodelling, pro-angiogenic and anti-angiogenic proteins, respectively. Y-axis represents the ratio of sample signal to reference signal, for each protein (n = 3). Error bars represent s.d.

The fibrin matrix initially provides structural support for platelets in order to form a stable hemostatic plug, but may also act as a natural carrier of cell-derived protein factors. While in nature these two functions are executed concurrently, here we used the HCM to differentially assess the property of the matrix as factor carrier without the presence of cells in it, thus exclusively analyzing the matrix bound/released proteome (i.e. separating this from the pool of cell-secreted unbound factors). Following a top-to-bottom approach, we first qualitatively and quantitatively investigated the proteomic composition of releasates from cell-free fibrin matrices, obtained after 7 days HCM culture, by employing mass spectrometry (note; inhibition of fibrinolysis during culture, through aprotinin present in the fibrinogen solution, minimized the subsequent loss of matrix-bound factors into culture media). We identified 281 proteins, which were sorted and plotted according to their intensity based absolute quantitation (IBAQ) values, from the most abundant protein on the left to the least abundant on the right (Fig 3A). Among these, we identified pro-angiogenic proteins (shown in green) such as matrix metalloproteinase 9 (MMP-9) and extracellular matrix protein (ECM-1), and anti-angiogenic factors (shown in red) such as antithrombin and alpha-2 antiplasmin, all of which are additionally depicted in two separate tables (Fig 3B). We further corroborated our mass spectrometry results with a semiquantitative analysis of fibrin matrix releasates using an angiogenesis-specific proteome array which revealed, in addition to the angiogenesis-related matrix remodelling proteins MMP-9,-8 and TIMP-1,-4, the presence of the pro-angiogenic factors angiogenin, IL8, endothelin-1 and EG-VEGF. Among the angiogenic inhibitors that could be detected, the most highly abundant were PF4, plasminogen/angiostatin, PEDF, endostatin and TSP1 (Fig 3C).

### The fibrin matrix binds pro- and anti-angiogenic factors differentially and mass-dependently

**Fig 4:**
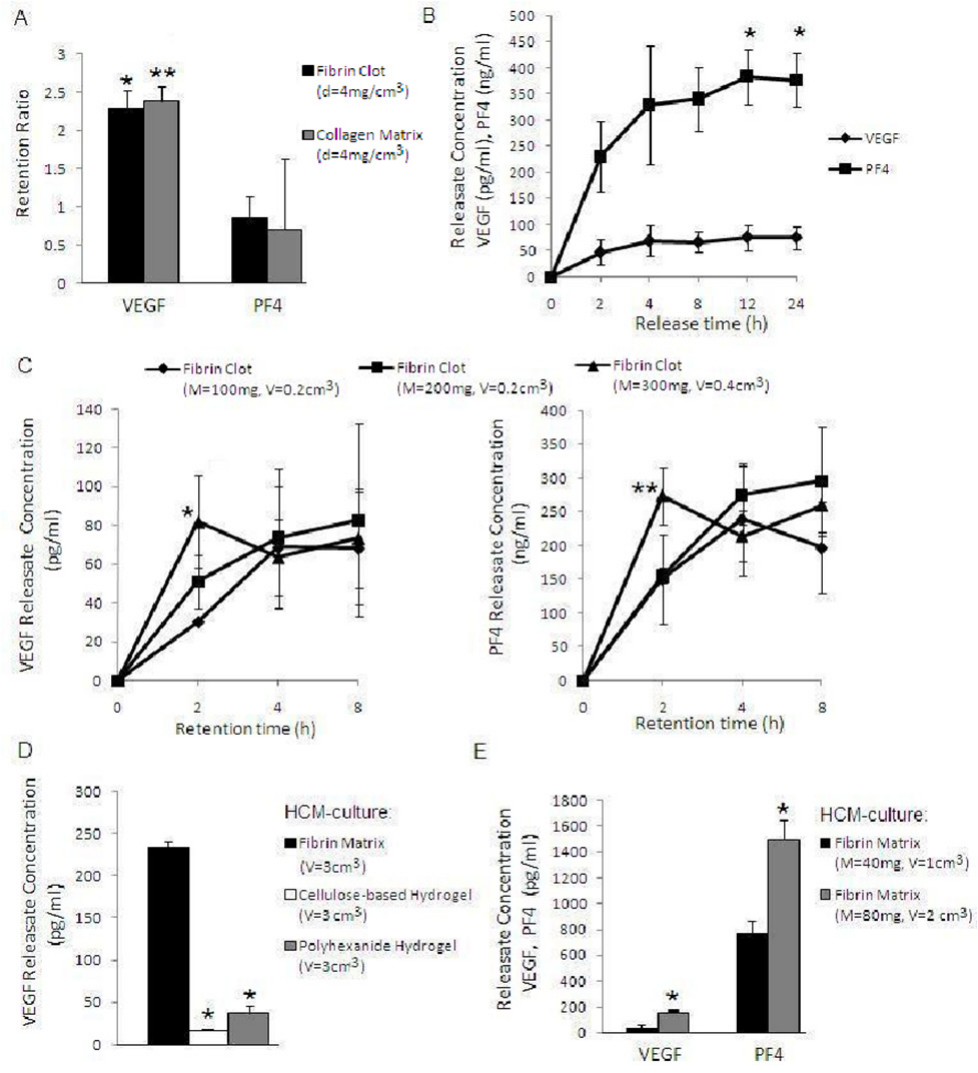
Differential and mass-dependent binding of VEGF and PF4 by the fibrin matrix. A) Plot comparing the VEGF and PF4 retention ratio (clot: plasma concentration) of cell-free fibrin clots (v = 1cm^3^) that were formed and incubated for 24h in plasma obtained from anticoagulated blood, after it had been cultured for 1h on collagen-coated substrates. Collagen matrices of comparable volume and protein density (v = 1cm^3^, d = 4 mg/cm^3^) were also tested as control in the same setup, *p<0.01 and **p<0.05 compared to corresponding PF4 value (n = 3). B) Plot comparing the VEGF and PF4 concentration in releasates of cell-free clots (total wet mass m = 300mg), that were formed and incubated for 24h in plasma derived from 7 day hypoxic blood culture. Afterwards clots were incubated in fresh medium and releasates were sampled every 2, 4, 8, 12 and 24h, *p<0.05 compared to 2h level (n = 3). C) Plots of VEGF (left) and PF4 (right) concentration in releasates of cell-free clots of varying total wet mass (M = 100, 200, 300mg). Clots were formed and incubated in plasma derived from 7 day hypoxic blood culture for 2, 4 and 8h, then centrifuged in fresh medium to obtain releasates, *p<0.05 compared to 100mg at 2h, **p<0.05 compared to 100 and 200mg at 2h (n = 3). D) Plot comparing the VEGF concentration in releasates obtained from equal volume (3cm^3^) fibrin matrices, cellulose-based hydrogels and polyhexanide hydrogels, that were harvested after 7 days HCM culture, *p<0.0001 compared to fibrin (n = 3). E) Plot comparing the VEGF and PF4 concentration in fibrin matrix releasates (concentrations were corrected for matrix volume-related dilution), obtained after 7 days HCM culture, for two fibrin(ogen) mass values (40 vs. 80mg, same matrix density = 0.04 g/cm^3^), *p<0.01 compared to fibrin 40mg (n = 3). Error bars represent s.d.

To quantitatively assess relative fibrin matrix binding of the key platelet-derived pro- and anti-angiogenic factors, VEGF and PF4, cell-free fibrin clots (v = 1cm^3^) were formed in situ by adding thrombin/Ca^2+^ to plasma derived from (anticoagulated) blood that had been cultured for 1h on collagen-coated substrates. Following 24h incubation of clots in plasma, the retention ratio (RR = clot: plasma concentration) for VEGF and PF4 was found to be ~2.5 and ~0.9, respectively (Fig 4A). In absolute terms, however, the amount of PF4 in clot releasates (402±73 ng/ml) was ~10^4^ greater than that of VEGF (49±1 pg/ml), reflecting the greater abundance of PF4 in plasma. Replacing the fibrin clots with collagen matrices of comparable protein density (v = 1cm^3^, d = 4 mg/cm^3^), in the same experimental setup, produced similar RR values for both factors (Fig 4A), implying that the density, i.e. the matrix mass-to-water ratio may be important for controlling protein factor retention, independent of matrix material.

To analyse the rate of factor release from the fibrin matrix, cell-free clots (total wet mass = 300mg) were formed by adding thrombin/Ca^2+^ to plasma that was derived from 7 day hypoxic blood culture, and incubated for 24h. As shown in Fig 4B, VEGF concentration in clot releasates reached a plateau within 4h, in contrast to 12h for PF4, indicating that VEGF was released somewhat faster than PF4.

While clot size predictably affects the concentration of platelet-only derived factors, such as PF4, proportionally to the number of trapped platelets (i.e. in a clot volume-driven manner), the concentration of VEGF and other factors that are also generated under hypoxia outside the clot should largely be determined through specific binding, and therefore depend on matrix fibrin mass to a greater extent. To test this hypothesis we investigated the effect of increasing the mass of cell-free clots, produced by adding an increasing amount of fibrinogen-thrombin/ Ca^2+^ to plasma derived from 7 day hypoxic blood culture, on the rate of factor retention. Clot saturation with both factors was observed within 8h, for all mass values tested (Fig 4C). VEGF binding, however, initially appeared to show greater mass-dependency since increasing clot (wet) mass from 100 to 300mg was correlated with a near-proportional (i.e. approx. 3-fold) increase in clot releasate VEGF concentration (30 vs. 82pg/ml) after 2h retention. At this time point, a mass-related increase in PF4 concentration (150 vs. 274ng/ml) was also observed, although this seemed to be more closely correlated with clot volume, which increased by 2-fold (0.2 vs. 0.4cm^3^) (note; binding of both factors was not limited by their availability in plasma, see Fig 4B).

As previously reported [9,11], and also suggested by these results, protein factor retention within the matrix is mediated through specific binding, but could also occur through passive trapping of factors within the inter-fibrillar water compartment (note; the matrix comprises mostly water). To specifically assess this in our culture model, we compared the VEGF concentration in releasates of HCM-cultured fibrin matrices with that in releasates of equal volume (3cm^3^) cellulose-based and polyhexanide hydrogel matrices comprising mostly water. As shown in Fig 4D, VEGF concentration in fibrin matrix releasates was ≥7 fold higher compared to hydrogel releasates (p<0.0001), thus indirectly confirming that the majority of VEGF within clots was matrix-bound.

Differential and mass-dependent binding of these factors by the matrix was next validated in the HCM model, where matrix fibrin mass was changed by varying the amount of fibrinogen (40 vs 80mg), while keeping the volumetric mass density constant (0.04 g/cm^3^). Analysis of matrix releasates obtained after 7 days HCM culture revealed that while doubling the fibrin(ogen) mass produced a ~3 to 5 fold increase in VEGF level (p<0.01), the correlation was weaker for PF4, which rose proportionally by ~2 fold (p<0.01) (Fig 4E). Therefore, in contrast to PF4, VEGF concentration rose above that predicted from the increase in matrix volume, confirming the higher mass-dependency of VEGF binding.

### Clot releasates induce distinct endothelial cell angiogenic responses

**Fig 5:**
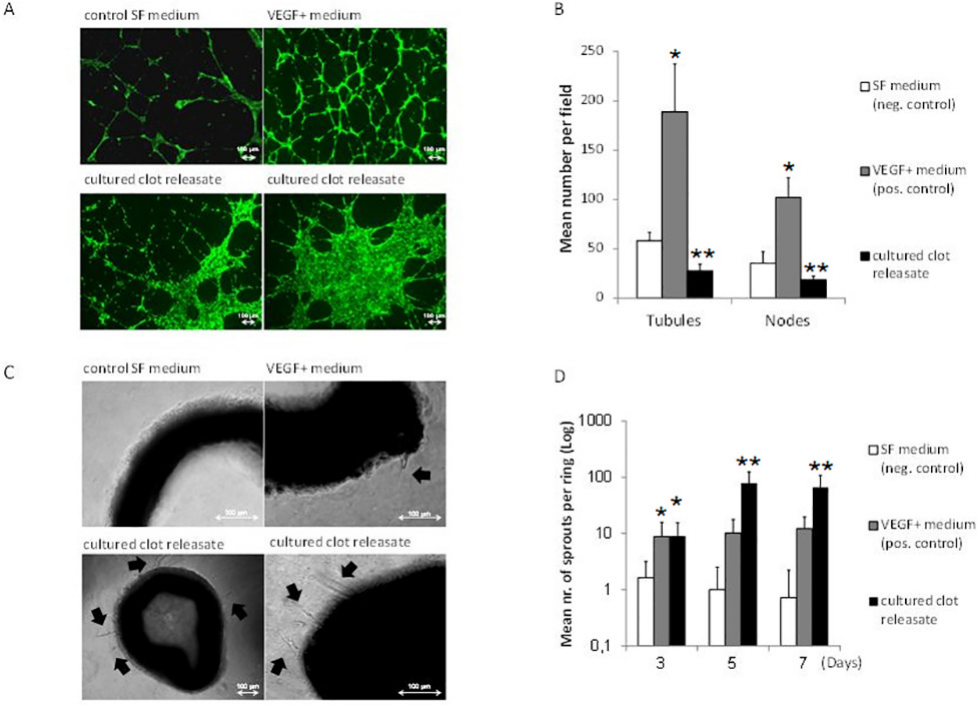
Releasates of cultured clots induce distinct endothelial cell angiogenic responses *in vitro*. A) Image panel showing the 16h tube formation by ECs seeded on matrigel, observed in response to releasates obtained from clots that were cultured on collagen matrices for 4 days at 37°C. Bars = 100 μm. B) Plot showing the mean number of tubules and nodes at 16h, *p<0.001 compared to neg.control and cultured clot releasate, **p<0.01 compared to neg.control. C) Image panel showing the 5 day sprout formation (arrowed) in the aortic ring-matrigel assay, observed in response to releasates obtained from cultured clots. Bars = 100 μm. D) Plot showing the mean number of sprouts over 7 days, *p<0.05 compared to neg.control, **p<0.05 compared to neg. and pos. control. SF medium and VEGF-containing SF medium were tested as negative and positive controls, respectively. For all assays n≥4. Error bars represent s.d.

To obtain a first impression of the angiogenic potential of factors released by clots into the hemostatic microenvironment, we cultured blood clots (in the presence of the derived serum) on collagen-coated matrices, at 37°C for 4 days, and sampled their releasates by centrifugation. When tested in an *in vitro* tube formation assay, cultured clot releasates generated a distinct angiogenic response compared to VEGF. As shown in Fig 5A and 5B, the number of tubules and nodes (i.e. tubule interconnections) formed by matrigel-seeded ECs at 16h in the presence of cultured clot releasates was significantly lower (p<0.001) than that obtained with VEGF-containing medium, while also failing to surpass the negative control baseline. Importantly, the morphology of the structures obtained was different, with significant endothelial cell clustering seen, instead of the regular nework of fine capillary-like structures induced by VEGF (Fig 5A). In contrast, the releasates of cultured clots appeared to support vessel sprouting in the aortic ring model (Fig 5C and 5D), to a greater extent than VEGF, suggesting that the biochemical regulation of this response differed from that of tubulogenesis.

### Changes in fibrin matrix mass facilitate differential regulation of EC migration, tube formation and sprouting

The results obtained so far suggest that differential protein binding allows the fibrin matrix to influence the balance of pro- and anti-angiogenic factors, through changes in its mass. This could in turn provide a means for differentially regulating the basic cellular processes involved in angiogenesis, namely EC chemotactic migration, new vessel formation and sprouting. To test this hypothesis, we investigated the effect of changes in the mass of HCM-harvested cell-free fibrin matrices (and with this releasate factor concentration) on these responses.

**Fig 6:**
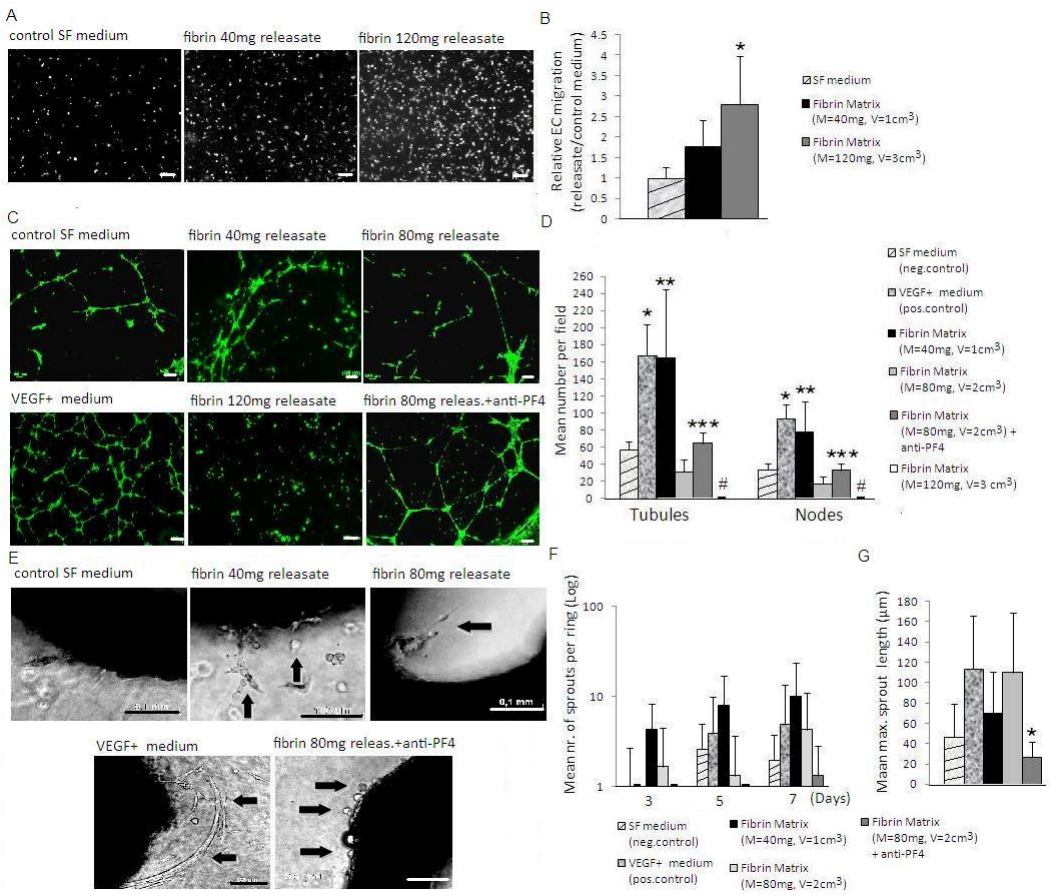
Changes in fibrin matrix mass differentially influence endothelial cell angiogenic responses. A) Image panel showing the 24h EC chemotactic migration through matrigel membrane, observed in response to releasates obtained from matrices of varying fibrin(ogen) mass (40 vs. 120mg). Bars = 100 μm. B) Plot showing the ratio of mean fluorescence intensity of migrating ECs at 24h in response to releasates relative to control medium, *p<0.01 compared to control medium. C) Image panel showing the 16h tube formation by ECs seeded on matrigel, observed in response to releasates obtained from matrices of varying fibrin(ogen) mass (40 vs 80 vs 120mg). Bars = 100 μm. D) Plot showing the mean number of tubules and nodes at 16h, *p<0.001 compared to all conditions except fibrin 40mg,**p<0.01 compared to all conditions except pos.control, ***p<0.01 compared to fibrin 80mg, #p<0.05 compared to all conditions except fibrin 80mg. E) Image panel showing the 5 day sprout formation (arrowed) in the aortic ring-matrigel assay, observed in response to releasates obtained from matrices of varying fibrin(ogen) mass (40 vs 80mg). Bars = 100 μm. F) Plot showing the mean number of sprouts per ring over 7 days. G) Plot showing the mean max. sprout length reached by day 5, *p<0.05 compared to pos.control and fibrin 80mg. Releasates were obtained from cell-free matrices that were harvested following 7 days HCM culture. Where indicated, releasates were incubated with anti-PF4 for 12h before testing. SF medium and VEGF-containing SF medium were tested as negative and positive controls, respectively. For all assays n≥4. Error bars represent s.d.

Increasing the matrix fibrin(ogen) mass from 40 to 120mg appeared to enhance the releasate-induced chemotactic migration of ECs through a matrigel membrane at 24h (Fig 6A and 6B), as expected from the accompanying mass-related increase in VEGF level (see Fig 4E). This difference, however, was only significant when compared to factor-free control medium (p<0.01). In contrast, there was an inverse correlation between matrix fibrin mass and the number of tubules and nodes formed by matrigel-seeded ECs at 16h. Already at 80mg, tube formation was downregulated to the level of negative control (Fig 6C and 6D), despite the increase in VEGF releasate concentration from 40 to 80mg. At 120mg, tube formation was even significantly lower than negative control (p<0.05), suggesting an active inhibition of angiogenesis (Fig 6C and 6D).

We asked if PF4 was responsible for this effect, since this was a consistently detected angiogenic inhibitor in releasates, and is a factor known to antagonize the action of VEGF [26,27]. Blocking PF4 activity in releasates of 80mg fibrin matrices with an anti-PF4 antibody resulted in a significant twofold increase in tubule number (p<0.01) (Fig 6C and 6D). Tube formation remained, however, significantly lower than VEGF-supplemented control medium (p<0.001), suggesting that the PF4 inhibition through anti-PF4 antibody was only partly effective, and/or that the observed inhibition was only partly mediated through PF4.

Lastly, we tested sprouting angiogenesis in the aortic ring model, a more complex response that relies on basement membrane degradation, EC migration, proliferation and assembly into microvessels [28]. Sprouting was induced earlier by fibrin matrix releasates than VEGF-supplemented control medium (3 vs 5 days), where single cell/fibroblast outgrowth was more extensive (Fig 6E and 6F). In agreement with the results of the tube formation assay, a trend towards an inverse correlation between fibrin mass (40 vs 80mg) and sprout number could be seen, although differences were not significant in this model (Fig 6E and 6F). Surprisingly, blocking PF4 activity with anti-PF4 appeared to inhibit, rather than enhance sprouting. Here, the few sprouts that did form were first seen at 5 days and were very short, comprising one or two ECs (Fig 6E, 6F and 6G). Taken together, these results indicated that while PF4 in releasates may exert an inhibitory effect on sprouting at higher concentrations, a minimum amount of the factor is nonetheless necessary for initiation of the process.

## Discussion

While the molecular players involved in the induction of post-wound angiogenesis are relatively well characterized [1,4,5], only little is yet known about the mechanism(s) that regulate where and, more enigmatically, when this occurs. Such precise spatiotemporal control is indeed difficult to envisage, given that following initiation of coagulation, a plethora of pro-and anti-angiogenic factors are released into the hemostatic microenvironment from the very onset [4,5], as also illustrated in this work. This inevitably calls for the presence of mechanisms for directing the cellular biochemical signaling that is derived within, as well as outside the clot, to ensure that the right factors act at the right place and time. In this study we investigated the hypothesis that this role is fulfilled by the fibrin matrix, through changes in its mass (i.e. the total concentration of fibrin within the wound) and consequently in the relative levels of factors that are bound and released over time. By employing HCM culture, we could selectively focus on the function of the matrix as a factor-carrier, independently of its commonly examined function as a cell-scaffold. The advantage of this approach is that it enables an exclusive analysis of the matrix bound/released proteome, which would not be possible with normal cellular fibrin clots, since their releasates contain the complete pool of matrix bound and unbound (i.e. directly secreted from cells) factors. Our findings demonstrate that endothelial cell angiogenic responses can be regulated through the matrix-defined biochemical milieu, that may be constituted distal to the site of endothelial cell attachment/invasion, rather than simply through the local interaction of cells with the fibrin matrix.

The finding that fibrin and collagen matrices of comparable density retained a similar proportion of plasma VEGF and PF4 hinted at the importance of matrix mass as a key tool with which the matrix could define and modify the ambient biochemical signature. Given the large, yet physiological size (i.e. 1–3cm^3^ [29]) of fibrin clots/collagen matrices used in this experiment the results obtained were, to a great extent, likely influenced by limitations in factor diffusion. Since diffusion of low molecular weight proteins through a hydrogel matrix occurs primarily via the water hydrating the polymer network [30], matrix density (i.e. mass-to-water ratio) evidently becomes an important parameter which, at these matrix sizes, appears to dominate material-dependent factor specific binding. Therefore, changes in fibrin matrix mass could facilitate regulation of angiogenic signaling through alterations in factor diffusivity, as well as availability of factor binding sites.

First, we could show that clot hypoxia potentiates the platelet-derived pro-angiogenic (VEGF) signaling generated through coagulation, in alignment with the results of previous studies [17,21]. Moreover, the severity/duration of pericellular hypoxia was found to correlate with blood cell seeding density, through an increase in O_2_ consumption (note; this effect is distinct, and additional to any effect of mass-related limitation in O_2_ diffusivity through the clot). This indicates that cell accumulation in the maturing wound bed or within a growing thrombus exacerbates the already compromised O_2_ supply, by increasing local aerobic demand. Through its function as a cell-scaffold, the fibrin matrix therefore plays a supportive role in cell-mediated hypoxic conditioning of the hemostastic microenvironment, which in turn renders it more angiogenic. This effect could be further potentiated though the simultaneous hypoxia-induced downregulation in the expression of anti-angiogenic factors, such as TSP1 [21,23,31], information that we here show can be registered within the matrix. Our findings demonstrate, however, that besides its role in hosting adhering/migrating cells, the matrix provides a platform for directly communicating to surrounding tissue the need for generation of compensatory angiogenesis, according to wound size or extent of vascular occlusion, through mass-dependent binding and releasing of pro-angiogenic factors. The ability of the fibrin matrix to relatively retain more of the available VEGF than PF4, and the higher sensitivity of VEGF releasate levels to changes in matrix mass, observed here, may indeed represent a strategy for counter-balancing the release of PF4 and other anti-angiogenic factors (e.g. TSP1) at significantly higher concentrations following coagulation. Importantly, we showed that an increase in matrix mass and VEGF releasate concentration had a net pro-chemotactic effect, despite the presence of these inhibitors. This suggests that through its factor-carrier function the fibrin matrix facilitates the formation of tunable chemoattractive gradients, which is crucial for directing angiogenesis towards the injured area [12].

What is perhaps more elusive is how immediate angiogenesis, supported by platelet-derived and hypoxia-induced positive regulators, can be locally halted until the clot has reached a stable state, which can accommodate ingrowth of neovessels without upsetting hemostasis. Considering that in the presence of angiogenic stimulators fibrin provides a favorable scaffold for sprout angiogenesis [6,7,14] (i.e. initiation of angiogenesis is theoretically possible from the onset of trauma) makes this question especially compelling. Here we show that the releasates of cultured clots produce a different morphological pattern of tubulogenesis compared to that induced by VEGF, with a dysregulation of the normal capillary network structure, despite being able to promote EC sprouting. The finding that the negative correlation observed between fibrin matrix mass and releasate-induced tube formation was, at least partly, mediated through PF4 strongly suggests that the matrix can exercise biochemical control on angiogenesis by balancing the relative concentrations of pro- and anti-angiogenic factors. Importantly, while our data confirm that fibrin matrix releasates can support EC sprouting, they also point to a more complex dependence of this process on PF4, since presence of the factor at low concentrations might be required for EC detachment from pre-existing vasculature [32], while higher concentrations inhibit cell proliferation and tubulogenesis [33–36]. We do note that in HCM experiments the fibrinogen concentration used to prepare fibrin matrices was deliberately higher than that normally encountered in plasma, due to the low response threshold of *in vitro* angiogenesis assays, which made observation of the postulated inhibition more challenging. Emphasis is therefore placed on the comparative scope of these findings, which indicate that deposition of fibrin at a wound or thrombotic site leads to increased binding and local release of inhibitors, negating the concomitant accumulation of stimulators. The significance of this capacity of the matrix to concentrate anti-angiogenic factors at effective doses is highlighted by the fact that platelet releasates are known to be, on balance, pro-angiogenic [35,37,38]. The resulting shift in equilibrium between pro- and anti-angiogenic factors towards the latter, in turn prevents rapid (i.e. premature) vascularization/destabilization of the clot, as previously speculated [5]. Our results also suggest that in addition to PF4, other inhibitors such as (cleaved) antithrombin, TSP1, PEDF and endostatin (all of which were detected in fibrin matrix releasates through mass spectrometry/proteome array) could be involved in this process.

A large body of evidence already exists on the mechanisms that mediate PF4’s anti-angiogenic action [27,33,34,36,39]. Here we move the focus from the cellular/molecular targets to the matrix, showing how it can provide a source of controlled release for this factor. In this context, crosstalk between cell signaling and the matrix could play an important role since PF4 has been reported to alter the structure of the fibrin network, by reducing its porosity [11], while it might also regulate matrix breakdown through inhibition of MMP expression by ECs [40]. It is therefore likely that the matrix derives its barrier properties through a combination of biochemical and biophysical control. Since these two dimensions are naturally intertwined, we chose to test the effect of soluble factors in matrix releasates on EC angiogenic induction rather than seeding ECs on fibrin matrices directly. This allowed us to study the biochemical regulation of angiogenesis without biophysical-related bias, arising for example through differences in fibrin structure (which is largely influenced by the conditions in which fibrin has been polymerized) [14,41] and fibrinolysis (note; naturally occurring fibrin(ogen) variants display different fibrinolytic sensitivity, which itself influences endothelial cell proliferation and tube formation [41,42]). Importantly, the fact that these responses could be observed on matrigel, a good mimic of the basement membrane [43], seems to suggest that ECs are inhibited in their native state (e.g. by released/soluble PF4 [39]), even before they contact the matrix. However, further work is needed to clarify this.

It is reasonable to postulate that these inhibitory effects are reversed with the initiation of fibrinolysis, through a reduction in the local pool of anti-angiogenic factors. Thus, besides its established function in permitting EC invasion into the matrix [14,15], fibrinolysis could be of paramount importance in signaling the right timing for starting angiogenesis, by enabling angiogenic *dis*inhibition. This could indeed be complemented by the release of pro-angiogenic fibrin fragments (e.g. FnE), that work synergistically with other pro-angiogenic factors (e.g. VEGF, bFGF) [44]. The results of this study also suggest that the regression of sprout angiogenesis accompanying collagen accumulation in the wound, during maturation of granulation tissue, may be largely independent of alterations in the fibrin matrix-defined growth factor profile. This is supportive of findings in our previous work, where the releasates of blood culture-derived, granulation tissue-like collagen matrices were shown to strongly promote sprouting in the aortic ring model [21]. Therefore, collagen-mediated inhibition of angiogenesis might instead be predominantly accomplished through differential modulation of integrin receptor expression in ECs, as previously reported [6,7].

**Fig 7:**
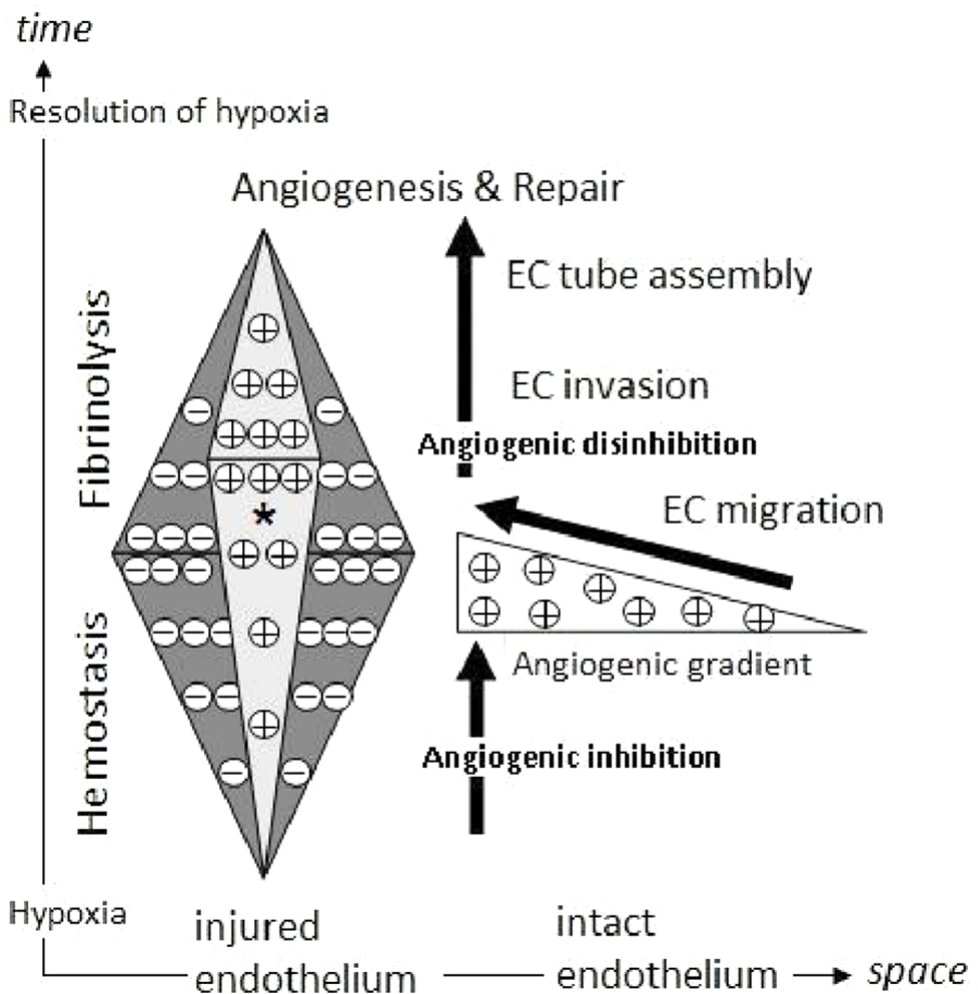
Proposed mechanism for matrix-dependent biochemical control of wound angiogenesis. A distinction is made here between the role of the matrix as cell-scaffold and factor-carrier. Through its carrier function, the fibrin matrix determines the balance of pro-and anti-angiogenic factors in the hemostatic microenvironment (light- and dark-grey triangles). Initially, anti-angiogenic factors (-) (e.g. PF4) are released by activated platelets and bound by the matrix at higher concentrations than pro-angiogenic factors (+) (e.g. VEGF). This inhibits neovessel formation around and into the clot, thus preventing clot destabilization and ensuring effective hemostasis. Meanwhile, increasing clot size and/or cell accumulation within the clot, under limited O_2_ supply (resulting from vascular injury), contributes to the development of local hypoxia. Hypoxia-induced upregulation of pro-angiogenic factor (e.g. VEGF) expression and downregulation of anti-angiogenic factor (e.g. TSP1) expression potentiates coagulation-mediated angiogenic signaling (*). Following completion of the hemostasic phase, the matrix undergoes controlled degradation through fibrinolysis, leading to depletion in the local pool of anti-angiogenic factors, while release of pro-angiogenic factors leads to the formation of chemoattractive gradients that direct endothelial cell (EC) migration towards the injured site. Angiogenic disinhibition enables vascularization of the matrix through fibrinolysis-mediated EC invasion, which facilitates resolution of hypoxia and tissue repair.

We put these findings in context of a proposed mechanism of matrix-dependent biochemical control, through which the fibrin matrix coordinates the balance between hemostasis and generation of spatiotemporally-defined angiogenesis (Fig 7). In this regard, the functionality of the matrix as protein factor-carrier not only supports, but rather dominates (and modulates) its role as cell-scaffold. Beyond providing a mechanistic model of how the key wound healing phases can operate in tandem, this also offers a theoretical framework for understanding, and potentially regulating other pathophysiological processes in which coagulation and angiogenesis are prominent features, such as the development of chronic hematomas [45,46], the association between elevated plasma fibrinogen and cardiovascular risk [47], the link between limited fibrin deposition and induction of angiogenesis in hemophilia [2], the formation of collateral circulation and clot recanalization/resolution following thrombotic/atherosclerotic vascular occlusion [17,36,48–50], the contribution of increased resistance of fibrin clots to degradation towards Alzheimer’s disease [51,52], and coagulopathy in malignant disease [4,53].

## Supporting Information

S1 Table Table with mass spectrometry preliminary data. Output files of raw data showing proteins of the fibrin matrix releasate identified via mass spectrometry, performed on samples where albumin-containing culture medium was used. Albumin is marked in yellow. In all subsequent experiments, albumin-free culture medium was used to reduce the interference with peptide detection.
(XLS)

S2 Table Table with full mass spectrometry analysis. Table showing proteins of the fibrin matrix releasate identified in at least one of the two biological replicates with at least one unique peptide via mass spectrometry. Pro-angiogenic proteins are marked green, anti-angiogenic proteins are marked red, while proteins involved in coagulation are written in blue. Under gene names the gene symbol can be found. Under protein identifiers the uniprot identifier of the respective protein is listed. Unique Peptides FibA and FibB indicate the identified peptides of each protein for the two biological replicates. Unprocessed output files can be found under supplementary data S3.
(DOC)

S3 Table Table with mass spectrometry output files (raw data). (XLS)
