# Supplementary material for: The Fibrin Matrix Regulates Angiogenic Responses within the Hemostatic Microenvironment through Biochemical Control
Source: PLoS One. 2015 Aug 28;10(8):e0135618. doi: 10.1371/journal.pone.0135618 (PMC4552838; doi:10.1371/journal.pone.0135618)
Supplement: S2 Table — Table showing proteins of the fibrin matrix releasate identified in at least one of the two biological replicates with at least one unique peptide via mass spectrometry. Pro-angiogenic proteins are marked green, anti-angiogenic proteins are marked red, while proteins involved in coagulation are written in blue. Under gene names the gene symbol can be found. Under protein identifiers the uniprot identifier of the respective protein is listed. Unique Peptides FibA and FibB indicate the identified peptides of each protein for the two biological replicates. Unprocessed output files can be found under supplementary data S3. (DOC) [file pone.0135618.s002.doc]

| **Protein names** | | **Gene names** | **Protein IDS** | **Unique peptides FibA** | **Unique peptides FibB** | |  |
| --- | --- | --- | --- | --- | --- | --- | --- |
| 1. Alpha-2-macroglobulin | | A2M | P01023 | 52 | 54 | |  |
| 1. Carboxypeptidase N catalytic chain | | CPN1 | P15169 | 2 | 2 | |  |
| 1. Carboxypeptidase N subunit 2 | | CPN2 | P22792 | 10 | 8 | |  |
| 1. Heparin cofactor 2 | | SERPIND1 | P05546 | 5 | 5 | |  |
| 1. Histidine-rich glycoprotein | | HRG | P04196 | 8 | 6 | |  |
| 1. Platelet glycoprotein Ib alpha chain | | GP1BA | P07359 | 5 | 5 | |  |
| 1. EGF-containing fibulin-like extracellular matrix protein 1 | | EFEMP1 | B4DW75 | 3 | 3 | |  |
| 1. Extracellular matrix protein 1 | | ECM1 | Q16610 | 8 | 8 | |  |
| 1. Fibrinogen alpha chain | | FGA | P02671 | 39 | 39 | |  |
| 1. Fibrinogen beta chain | | FGB | P02675 | 16 | 16 | |  |
| 1. Fibrinogen gamma chain | | FGG | P02679-2 | 18 | 18 | |  |
| 1. Fibronectin | | FN1 | P02751 | 94 | 99 | |  |
| 1. Matrix metalloproteinase-9 | | MMP9 | P14780 | 11 | 11 | |  |
| 1. Tenascin | | TNC | E9PC84 | 2 | 2 | |  |
| 1. Alpha-2-antiplasmin | | SERPINF2 | P08697 | 8 | 8 | |  |
| 1. Antithrombin-III | | SERPINC1 | P01008 | 10 | 10 | |  |
| 1. Kininogen-1 | | KNG1 | P01042-2 | 15 | 14 | |  |
| 1. Plasminogen | | PLG | P00747 | 35 | 35 | |  |
| 1. Protein Z-dependent protease inhibitor | | SERPINA10 | Q9UK55 | 11 | 10 | |  |
| 1. Prothrombin | | F2 | P00734 | 22 | 22 | |  |
| 1. Thrombospondin-1 | | THBS1 | P07996 | 6 | 6 | |  |
| 1. Vitamin K-dependent protein C | | PROC | P04070 | 6 | 5 | |  |
| 1. Fibulin-1 | | FBLN1 | P23142 | 8 | 8 | |  |
| 1. Fibulin-1 | | FBLN1 | P23142-4 | 1 | 6 | |  |
| 1. Pigment epithelium-derived factor | | SERPINF1 | P36955 | 7 | 6 | |  |
| 1. Platelet factor 4 | | PF4;PF4V1 | P02776 | 1 | 1 | |  |
| 1. Apolipoprotein B-100 | | APOB | P04114 | 13 | 22 | |  |
| 1. Beta-2-glycoprotein 1 | | APOH | P02749 | 5 | 5 | |  |
| 1. Coagulation factor IX | | F9 | P00740 | 7 | 6 | |  |
| 1. Coagulation factor V | | F5 | P12259 | 3 | 2 | |  |
| 1. Coagulation factor X | | F10 | P00742 | 11 | 11 | |  |
| 1. Coagulation factor XII | | F12 | P00748 | 2 | 2 | |  |
| 1. Coagulation factor XIII A chain | | F13A1 | P00488 | 14 | 15 | |  |
| 1. Coagulation factor XIII B chain | | F13B | P05160 | 22 | 22 | |  |
| 1. Hemoglobin subunit beta | | HBB | P68871 | 5 | 7 | |  |
| 1. Plasma kallikrein | | KLKB1 | P03952 | 3 | 5 | |  |
| 1. Plasma protease C1 inhibitor | | SERPING1 | B4E1H2 | 16 | 16 | |  |
| 1. Vitamin K-dependent protein S | | PROS1 | G5E9F8 | 8 | 9 | |  |
| 1. Vitamin K-dependent protein Z | | PROZ | P22891 | 3 | 4 | |  |
| 1. Vitronectin | | VTN | P04004 | 11 | 11 | |  |
| 1. von Willebrand factor | | VWF | P04275 | 84 | 84 | |  |
| 1. 78 kDa glucose-regulated protein | HSPA5 | | P11021 | 0 | | 1 | |
| 1. Afamin | AFM | | P43652 | 20 | | 21 | |
| 1. Alpha-1-acid glycoprotein 1 | ORM1 | | P02763 | 6 | | 6 | |
| 1. Alpha-1-acid glycoprotein 2 | ORM2 | | P19652 | 4 | | 4 | |
| 1. Alpha-1-antichymotrypsin | SERPINA3 | | P01011 | 4 | | 4 | |
| 1. Alpha-1-antitrypsin | SERPINA1 | | P01009 | 26 | | 26 | |
| 1. Alpha-1B-glycoprotein | A1BG | | P04217 | 9 | | 9 | |
| 1. Alpha-2-HS-glycoprotein | AHSG | | P02765 | 6 | | 6 | |
| 1. Angiotensinogen | AGT | | P01019 | 8 | | 7 | |
| 1. Apolipoprotein A-I | APOA1 | | P02647 | 14 | | 14 | |
| 1. Apolipoprotein D | APOD | | P05090 | 5 | | 5 | |
| 1. Apolipoprotein E | APOE | | P02649 | 3 | | 5 | |
| 1. Apolipoprotein(a) | LPA | | P08519 | 2 | | 2 | |
| 1. Attractin | ATRN | | O75882-3 | 6 | | 6 | |
| 1. Band 3 anion transport protein | SLC4A1 | | P02730 | 0 | | 9 | |
| 1. Basement membrane-specific heparan sulfate proteoglycan core protein | HSPG2 | | P98160 | 6 | | 6 | |
| 1. Biotinidase | BTD | | C9JSN9 | 1 | | 1 | |
| 1. Bisphosphoglycerate mutase | BPGM | | P07738 | 0 | | 1 | |
| 1. C4b-binding protein alpha chain | C4BPA | | P04003 | 15 | | 14 | |
| 1. CD44 antigen | CD44 | | H0YDX6 | 2 | | 2 | |
| 1. Ceruloplasmin | CP | | P00450 | 23 | | 23 | |
| 1. Clusterin | CLU | | P10909-4 | 9 | | 9 | |
| 1. Collagen alpha-1(I) chain | COL1A1 | | P02452 | 12 | | 13 | |
| 1. Collagen alpha-2(I) chain | COL1A2 | | P08123 | 7 | | 7 | |
| 1. Complement C1q subcomponent subunit A | C1QA | | P02745 | 2 | | 2 | |
| 1. Complement C1q subcomponent subunit B | C1QB | | D6RA08 | 6 | | 6 | |
| 1. Complement C1q subcomponent subunit C | C1QC | | P02747 | 4 | | 4 | |
| 1. Complement C1r subcomponent | C1R | | P00736 | 1 | | 4 | |
| 1. Complement C1r subcomponent-like protein | C1RL | | Q9NZP8 | 1 | | 1 | |
| 1. Complement C1s subcomponent | C1S | | P09871 | 9 | | 8 | |
| 1. Complement C3 | C3 | | P01024 | 84 | | 82 | |
| 1. Complement C4-A | C4A | | P0C0L4 | 6 | | 5 | |
| 1. Complement C4-B | C4B | | P0C0L5 | 5 | | 4 | |
| 1. Complement C5 | C5 | | P01031 | 32 | | 36 | |
| 1. Complement component C6 | C6 | | P13671 | 14 | | 14 | |
| 1. Complement component C7 | C7 | | P10643 | 2 | | 3 | |
| 1. Complement component C8 alpha chain | C8A | | P07357 | 4 | | 4 | |
| 1. Complement component C8 beta chain | C8B | | F5GY80 | 2 | | 3 | |
| 1. Complement component C9 | C9 | | P02748 | 5 | | 6 | |
| 1. Complement factor B | CFB | | B4E1Z4 | 29 | | 29 | |
| 1. Complement factor H | CFH | | P08603 | 37 | | 40 | |
| 1. Complement factor H-related protein 1 | CFHR1 | | Q03591 | 2 | | 2 | |
| 1. Complement factor H-related protein 2 | CFHR2 | | P36980-2 | 1 | | 1 | |
| 1. Complement factor H-related protein 5 | CFHR5 | | Q9BXR6 | 1 | | 1 | |
| 1. Complement factor I | CFI | | G3XAM2 | 2 | | 2 | |
| 1. Corneodesmosin | CDSN | | Q15517 | 0 | | 1 | |
| 1. Dermcidin | DCD | | P81605 | 4 | | 4 | |
| 1. Desmocollin-1 | DSC1 | | Q08554-2 | 1 | | 2 | |
| 1. Endoplasmin | HSP90B1 | | P14625 | 2 | | 3 | |
| 1. Ficolin-3 | FCN3 | | O75636-2 | 1 | | 1 | |
| 1. Galectin-3-binding protein | LGALS3BP | | Q08380 | 5 | | 5 | |
| 1. Glutaminyl-peptide cyclotransferase | QPCT | | B5MCZ9 | 1 | | 2 | |
| 1. Haptoglobin | HP | | P00738 | 9 | | 9 | |
| 1. Hemoglobin subunit alpha | HBA1;HBA2 | | P69905 | 3 | | 4 | |
| 1. Hemopexin | HPX | | P02790 | 11 | | 12 | |
| 1. Ig alpha-1 chain C region | IGHA1 | | P01876 | 3 | | 4 | |
| 1. Ig gamma-1 chain C region | IGHG1 | | P01857 | 7 | | 8 | |
| 1. Ig gamma-2 chain C region | IGHG2 | | P01859 | 2 | | 2 | |
| 1. Ig gamma-3 chain C region | IGHG3 | | P01860 | 3 | | 3 | |
| 1. Ig gamma-4 chain C region | IGHG4 | | P01861 | 1 | | 1 | |
| 1. Ig heavy chain V-I region HG3 |  | | P01743 | 1 | | 1 | |
| 1. Ig heavy chain V-II region ARH-77 |  | | P06331 | 2 | | 2 | |
| 1. Ig heavy chain V-II region WAH |  | | P01824 | 2 | | 1 | |
| 1. Ig heavy chain V-III region BRO |  | | P01766 | 1 | | 1 | |
| 1. Ig heavy chain V-III region BUR |  | | P01773 | 1 | | 1 | |
| 1. Ig heavy chain V-III region BUT |  | | P01767 | 1 | | 1 | |
| 1. Ig heavy chain V-III region GAL |  | | P01781 | 3 | | 3 | |
| 1. Ig heavy chain V-III region HIL |  | | P01771 | 1 | | 1 | |
| 1. Ig heavy chain V-III region TIL |  | | P01765 | 0 | | 0 | |
| 1. Ig heavy chain V-III region TUR |  | | P01779 | 1 | | 1 | |
| 1. Ig heavy chain V-III region VH26 |  | | P01764 | 1 | | 1 | |
| 1. Ig kappa chain V-I region AG |  | | P01593 | 0 | | 0 | |
| 1. Ig kappa chain V-I region BAN |  | | P04430 | 1 | | 1 | |
| 1. Ig kappa chain V-I region EU |  | | P01598 | 2 | | 2 | |
| 1. Ig kappa chain V-I region Lay |  | | P01605 | 1 | | 1 | |
| 1. Ig kappa chain V-I region Ni |  | | P01613 | 1 | | 1 | |
| 1. Ig kappa chain V-I region Scw |  | | P01609 | 0 | | 1 | |
| 1. Ig kappa chain V-I region Wes |  | | P01611 | 1 | | 1 | |
| 1. Ig kappa chain V-II region TEW |  | | P01617 | 2 | | 2 | |
| 1. Ig kappa chain V-III region VG |  | | P04433 | 1 | | 1 | |
| 1. Ig kappa chain V-III region WOL |  | | P01623 | 3 | | 3 | |
| 1. Ig kappa chain V-IV region Len |  | | P01625 | 1 | | 1 | |
| 1. Ig lambda chain V-I region HA |  | | P01700 | 2 | | 2 | |
| 1. Ig lambda chain V-I region NEW |  | | P01701 | 2 | | 2 | |
| 1. Ig lambda chain V-III region LOI |  | | P80748 | 2 | | 2 | |
| 1. Ig lambda chain V-III region SH |  | | P01714 | 2 | | 2 | |
| 1. Ig lambda chain V-IV region Bau |  | | P01715 | 1 | | 1 | |
| 1. Ig lambda chain V-IV region Hil |  | | P01717 | 1 | | 1 | |
| 1. Ig lambda chain V-IV region MOL |  | | P06889 | 1 | | 1 | |
| 1. Ig mu chain C region | IGHM | | P01871 | 13 | | 13 | |
| 1. Immunoglobulin J chain | IGJ | | D6RHJ6 | 2 | | 2 | |
| 1. Insulin-like growth factor-binding protein complex acid labile subunit | IGFALS | | P35858 | 0 | | 5 | |
| 1. Inter-alpha-trypsin inhibitor heavy chain H1 | ITIH1 | | P19827 | 10 | | 10 | |
| 1. Inter-alpha-trypsin inhibitor heavy chain H2 | ITIH2 | | Q5T985 | 27 | | 27 | |
| 1. Inter-alpha-trypsin inhibitor heavy chain H3 | ITIH3 | | Q06033-2 | 22 | | 22 | |
| 1. Inter-alpha-trypsin inhibitor heavy chain H4 | ITIH4 | | Q14624-3 | 14 | | 17 | |
| 1. Lactotransferrin | LTF | | E7EQB2 | 13 | | 15 | |
| 1. Leucine-rich alpha-2-glycoprotein | LRG1 | | P02750 | 2 | | 2 | |
| 1. Lumican | LUM | | P51884 | 4 | | 4 | |
| 1. Myeloperoxidase | MPO | | P05164-2 | 2 | | 2 | |
| 1. N-acetylmuramoyl-L-alanine amidase | PGLYRP2 | | Q96PD5 | 3 | | 2 | |
| 1. Neural cell adhesion molecule 1 | NCAM1 | | R4GMN9 | 3 | | 3 | |
| 1. Neuropilin-1 | NRP1 | | Q5JWQ4 | 2 | | 0 | |
| 1. Neutrophil defensin 3 | DEFA3;DEFA1 | | P59666 | 2 | | 2 | |
| 1. Neutrophil gelatinase-associated lipocalin | LCN2 | | P80188-2 | 2 | | 2 | |
| 1. Plasma serine protease inhibitor | SERPINA3;SERPINA5 | | G3V5I3 | 1 | | 1 | |
| 1. Prolactin-inducible protein | PIP | | P12273 | 2 | | 2 | |
| 1. Protein AMBP | AMBP | | P02760 | 9 | | 11 | |
| 1. Proteoglycan 4 | PRG4 | | Q92954-3 | 1 | | 2 | |
| 1. Retinol-binding protein 4 | RBP4 | | Q5VY30 | 7 | | 9 | |
| 1. Serotransferrin | TF | | P02787 | 28 | | 28 | |
| 1. Serum amyloid P-component | APCS | | P02743 | 3 | | 3 | |
| 1. Serum paraoxonase/arylesterase 1 | PON1 | | P27169 | 4 | | 4 | |
| 1. Sex hormone-binding globulin | SHBG | | I3L2X4 | 1 | | 1 | |
| 1. Sushi, von Willebrand factor type A, EGF and pentraxin domain-containing protein 1 | SVEP1 | | Q4LDE5-3 | 0 | | 1 | |
| 1. Tenascin-X | TNXB | | P22105-3 | 4 | | 5 | |
| 1. Tetranectin | CLEC3B | | E9PHK0 | 1 | | 1 | |
| 1. Transthyretin | TTR | | P02766 | 10 | | 10 | |
| 1. Vitamin D-binding protein | GC | | P02774 | 11 | | 12 | |
| 1. Zinc-alpha-2-glycoprotein | AZGP1 | | P25311 | 13 | | 13 | |

**S2 Table 2.** **Table with full mass spectrometry analysis.** Table showing proteins of the fibrin matrix releasate identified in at least one of the two biological replicates with at least one unique peptide via mass spectrometry. Pro-angiogenic proteins are marked green, anti-angiogenic proteins are marked red, while proteins involved in coagulation are written in blue. Under gene names the gene symbol can be found. Under protein identifiers the uniprot identifier of the respective protein is listed. Unique Peptides FibA and FibB indicate the identified peptides of each protein for the two biological replicates. Unprocessed output files can be found under supplementary data S3.
